# Role of liver FGF21-KLB signaling in ketogenic diet-induced amelioration of hepatic steatosis

**DOI:** 10.1038/s41387-024-00277-3

**Published:** 2024-04-12

**Authors:** Wanrong Guo, Huanyi Cao, Yunfeng Shen, Wuguo Li, Wei Wang, Lidan Cheng, Mengyin Cai, Fen Xu

**Affiliations:** 1 Department of Endocrinology and Metabolism, The Third Affiliated Hospital of Sun Yat-sen University, Guangzhou, China; 2 Guangdong Provincial Key Laboratory of Diabetology, Guangzhou, China; 3 Guangzhou Municipal Key Laboratory of Mechanistic and Translational Obesity Research, The Third Affiliated Hospital of Sun Yat-sen University, Guangzhou, China; 4 Medical Intensive Care Unit, The Third Affiliated Hospital of Sun Yat-sen University, Guangzhou, China; 5 Department of Endocrinology, Guangdong Provincial People’s Hospital, Guangdong Academy of Medical Sciences, Southern Medical University, Guangzhou, China; 6 Department of Endocrinology and Metabolism, The Second Affiliated Hospital of Nanchang University, Nanchang, China; 7 Animal Experiment Center, The First Affiliated Hospital of Sun Yat-sen University, Guangzhou, China

**Keywords:** Metabolic syndrome, Obesity

## Abstract

**Background:**

The effectiveness of ketogenic diet (KD) in ameliorating fatty liver has been established, although its mechanism is under investigation. Fibroblast growth factor 21 (FGF21) positively regulates obesity-associated metabolic disorders and is elevated by KD. FGF21 conventionally initiates its intracellular signaling via receptor β-klotho (KLB). However, the mechanistic role of FGF21-KLB signaling for KD-ameliorated fatty liver remains unknown. This study aimed to delineate the critical role of FGF21 signaling in the ameliorative effects of KD on hepatic steatosis.

**Methods:**

Eight-week-old C57BL/6 J mice were fed a chow diet (CD), a high-fat diet (HFD), or a KD for 16 weeks. Adeno-associated virus-mediated liver-specific KLB knockdown mice and control mice were fed a KD for 16 weeks. Phenotypic assessments were conducted during and after the intervention. We investigated the mechanism underlying KD-alleviated hepatic steatosis using multi-omics and validated the expression of key genes.

**Results:**

KD improved hepatic steatosis by upregulating fatty acid oxidation and downregulating lipogenesis. Transcriptional analysis revealed that KD dramatically activated FGF21 pathway, including KLB and fibroblast growth factor receptor 1 (FGFR1). Impairing liver FGF21 signaling via KLB knockdown diminished the beneficial effects of KD on ameliorating fatty liver, insulin resistance, and regulating lipid metabolism.

**Conclusion:**

KD demonstrates beneficial effects on diet-induced metabolic disorders, particularly on hepatic steatosis. Liver FGF21-KLB signaling plays a critical role in the KD-induced amelioration of hepatic steatosis.

## Introduction

Non-alcoholic fatty liver disease (NAFLD) stands as one of the most common chronic liver diseases globally, with an estimated prevalence of 32.4% [1]. Lifestyle interventions, including dietary adjustments and physical activity, are the main recommendations for managing NAFLD. Within this context, caloric intake, dietary composition, and meal timing have emerged as focal points in NAFLD treatment [2].

The ketogenic diet (KD), initially developed for epilepsy treatment, is characterized by a composition with over 90% of calories from fat and less than 1% of calories from carbohydrates [3]. Clinical studies have demonstrated that KD induced a remarkable reduction in body weight and the amelioration of fatty liver, accompanied by decreased alanine aminotransferase and aspartate aminotransferase levels [4–6]. Rodent models fed a KD have showcased weight loss, improved insulin sensitivity, lipid metabolism, and glucose metabolism [7–10]. In hepatology studies, KD has been noted to enhance hepatic energy expenditure, promote fatty acid oxidation, and suppress lipogenesis compared with high-fat diets (HFD) or western diets [7, 8]. Mechanistically, KD activates AMP-activated protein kinase and inhibits acetyl-CoA carboxylase (ACC) activity in the liver and muscle [7]. Moreover, our previous study has elucidated that KD reduces malonylation of acetyl-CoA carboxylase 1 (ACC1), critical for activity and stability of ACC1, thereby alleviating fatty liver [11]. Besides the liver, KD activates brown adipose tissue (BAT) function by upregulating heat-related gene expression [12] and mitigates inflammation in white adipose tissues (WATs) [13].

Fibroblast growth factor 21 (FGF21), a crucial metabolic regulator primarily synthesized in the liver, modulates the metabolism of multiple tissues [14]. Elevating serum FGF21 levels through exogenous administration or liver overexpression induces weight loss, enhances energy expenditure, improves glucose metabolism and insulin sensitivity, alleviates hepatic steatosis, promotes lipolysis, induces browning of WAT, and activates BAT function [15–21]. A phase 2a clinical trial has further validated the beneficial effects of FGF21 on steatohepatitis [22].

FGF21 is upregulated by KD [23, 24]. FGF21-deficient mice fed a KD gained weight and developed hepatic steatosis, whereas control mice exhibited weight loss and reduced fatty liver [25]. Liver-specific FGF21-knockdown mice fed a KD also displayed fatty liver compared with control mice [23]. FGF21 signals target cells through a receptor complex composed of FGF receptor (FGFR), FGFR1c, and a co-receptor called β-klotho (KLB). Elimination of either FGFR1 or KLB impairs the acute insulin-sensitizing effects of FGF21 [26, 27]. Thus, we hypothesized that KD might ameliorate hepatic steatosis via hepatic FGF21-KLB signaling. In this study, we aimed to delineate the critical role of FGF21 signaling in the ameliorative effects of KD on hepatic steatosis in order to offer new insights into the potential of KD in the treatment of NAFLD.

## Materials and methods

### Animal experiments

Seven-week-old male C57BL/6 J mice were purchased from GemPharmatech (Nanjing, China). Mice were housed in a standard specific pathogen-free facility with free access to food and water. After a 1-week acclimatization period, eight-week-old mice were used in two experiments. In the first experiment, mice were divided randomly into three groups (*n* = 5 per group), which were exposed to a chow diet (CD, 11% fat [kcal%]; Guangdong Medical Laboratory Animal Center, Guangzhou, China), an HFD (58% fat [kcal%]; D12331; Research Diets, New Brunswick, NJ, USA), or an HFD and a ketogenic diet (KD, 90.5% fat [kcal%], TD.160153, Envigo, USA) alternating every 2 weeks for a total period of 16 weeks. In the second experiment, adeno-associated virus (AAV) expressing short-hairpin RNAs targeting *β-klotho* (sh*Klb*) and negative control (sh*Ctrl*) were administered to generate liver-specific KLB knockdown mice and control mice, and then mice were subjected to 16-week diet intervention on the next day. Body weight, blood glucose, and food intake were recorded every 2 weeks. Blood and tissue samples were collected after overnight fasting. All animal experiments complied with the ARRIVE guidelines and were approved by the Institutional Animal Care and Use Committee of Sun Yat-sen University.

### AAV9-mediated gene knockdown

AAVs expressing sh*Klb* (5′-GCAATCTGTCCAAAGTTAACA-3′) and sh*Ctrl* were purchased from Genechem Co., Ltd (Shanghai, China). The sequence was validated in a previous study to effectively knock down *Klb* expression in mouse liver [20]. AAV9 (1.0 × 10^11^ active viral particles/mouse) was injected via the tail vein to suppress *Klb* expression in the liver.

### Body composition measurement

After the dietary exposure, body composition was measured by a quantitative magnetic resonance EchoMRI™-100 (EchoMRI LLC, Houston, TX, USA) consciously according to the manufacturer’s instructions. Mice were carefully fixed in tubes and then inserted into the EchoMRI™-100 machine one at a time, and fat and lean masses were analyzed and recorded by the machine within 1 min. Fat mass is presented as the ratio to body weight.

### Glucose and insulin tolerance tests

Glucose tolerance test was performed in overnight-fasted mice by intraperitoneally injecting with glucose (2.0 g/kg body weight). Blood samples were collected for serum insulin measurement. For the insulin tolerance test, the mice were intraperitoneally injected with insulin (0.65 units/kg body weight, Novolin R, Novo Nordisk Inc., Denmark) after 6 h of fasting. Blood glucose levels were recorded at various time points after the injection.

### Biochemical and immunological analyses

Triglycerides, total cholesterol, and free fatty acids were quantified using commercial kits (triglycerides, K622-100; cholesterol, K603-100; free fatty acids, K612-100; Biovision, Milpitas, CA, USA). Serum insulin concentration was measured with an ELISA kit (#10-1247-01; Mercodia, Uppsala, Sweden), and another commercial kit (#32180; Immunodiagnostics Limited, Science Park, Hong Kong, China) was used in FGF21 measurement. Every sample was assayed in duplicate.

### Hematoxylin and eosin staining

Hematoxylin and eosin staining was performed according to standard procedures [28]. Fresh tissues were fixed in 4% paraformaldehyde solution (GBCBIO, Guangzhou, China) at 4°C overnight. Then, tissues were embedded in paraffin and sectioned at 3–5 μm at room temperature. The sections were deparaffinized twice with fresh xylene and dehydrated with ethanol at a gradient concentration. Next, the nucleus was stained with hematoxylin, and the cytoplasm was stained with eosin. Photomicrographs were obtained using a DMi8 inverted microscope (Leica Microsystems, Wetzlar, Germany).

### Oil Red O staining

Oil Red O staining was performed according to standard procedures [28, 29]. Briefly, fresh liver tissues were embedded in OCT compound (Sakura Finetek, Torrance, CA, USA) and stored at −80°C. Liver tissues were next sectioned at 6–8 μm at −18 °C and washed with 60% isopropanol. A stock solution was prepared using Oil Red O powder (O0625; Sigma Aldrich, St Louis, MO, USA) dissolved in isopropanol and protected from light; a working concentration of 60% was diluted with ddH_2_O. The sections were stained with Oil Red O working solution for 1 h and washed under running tap water for 20 min. After Oil Red O staining, tissue sections were counterstained with hematoxylin for 1 min. The Oil Red O staining procedure was performed in the dark. Photomicrographs were obtained using a DMi8 inverted microscope (Leica Microsystems).

### Real-time qPCR

Total RNA was extracted from frozen tissues using Trizol Reagent (Sigma Aldrich) [30]. cDNA was synthesized from the RNA by reverse transcription using a Transcriptor First Strand cDNA Synthesis Kit (Roche Applied Science, Basel, Switzerland). Real-time PCR was performed using a LightCycler 480 System with LightCycler 480 SYBR Green Master Mix (Roche Applied Science). Target gene expression was normalized against β-actin, and the fold change in mRNA expression was determined using the 2^-ΔΔCT^ method.

### Western blotting

Protein was extracted from tissues using RIPA lysis and extraction buffer (89900; Thermo Fisher Scientific, Waltham, MA, USA) combined with Halt™ Protease and Phosphatase Inhibitor Cocktail (78440; Thermo Fisher Scientific). Next, an equal amount (30 μg) of protein was separated by 10% SDS-PAGE and transferred onto a polyvinylidene fluoride membrane (EMD Millipore, Burlington, MA, USA). Membranes were incubated with 0.1% (1:1000) target primary antibodies at 4°C overnight and then with 0.01% (1:10000) secondary antibodies at room temperature for 1 h protected from light. After washing thrice with TBS, the protein bands were visualized using the Odyssey Infrared Imaging System (LI-COR, Nebraska, USA). Antibodies against acetyl-CoA carboxylase (ACC) (3676 S), fatty acid synthase (FASN) (3180 S), stearyl-coenzyme A desaturase 1 (SCD1) (2794 S), and β-actin (4970 s) were purchased from Cell Signaling Technology (Danvers, MA, USA). Fibroblast growth factor receptor 1 (FGFR1) (60325-1-Ig) antibody was purchased from Proteintech (Wuhan, China). Carnitine palmitoyltransferase 1α (CPT1α) antibody (sc-31128) was purchased from Santa Cruz Biotechnology (Dallas, TX, USA). KLB antibody (AF-2619) was purchased from R&D System (Minneapolis, MN, USA).

### Immunofluorescence staining

Immunofluorescence staining of mouse livers was performed as previously described [11]. Tissue sections were prepared for hematoxylin and eosin staining as previously described. Sections were immersed in preheated citrate buffer and blocked with 3% hydrogen peroxide. Primary antibodies (0.5%, 1:200) against ACC, FASN, CPT1α, FGFR1, and KLB were applied to the sections overnight at 4°C, followed by incubation with secondary antibodies (0.2%, 1:500). Finally, the nuclei were stained with DAPI for 10 min and washed with PBS. Photomicrographs were obtained using a DMi8 inverted microscope and Leica Qwin image analysis software (Leica Microsystems).

### RNA sequencing

Total RNA was extracted from fresh mouse livers using an RNeasy Plus Mini kit (QIAGEN, Hilden, Germany) according to the manufacturer’s instructions. An Agilent 2100 Bioanalyzer System (Agilent Technologies, Palo Alto, CA, USA) and Agilent RNA 6000 Nano Kit (Agilent Technologies) were used to assess the integrity and concentration of total RNA. Samples with an RNA integrity number >8 were considered suitable for further analyses. RNA sequencing and data analysis (GEO: GSE226139) were conducted by Pan-Guarantee Biotechnology Co., Ltd (Guangzhou, China), as previously described [11].

### Proteomic analysis

Proteins from mouse livers were isolated using a lysis buffer (8 mol/L urea, 1% protease inhibitor cocktail, 3 mmol/L trichostatin A, and 50 mmol/L nicotinamide), followed by sonication using a high-intensity ultrasonic processor (Scientz Biotechnology, Ningbo, China) and centrifugation. A BCA kit (Thermo Fisher Scientific) was used to measure the protein concentration according to the manufacturer’s instructions. Proteomic analysis (PRIDE: PXD040481) was performed using the Jingjie PTM Bio-Labs (Hangzhou, China) as previously described [11].

### Statistical analyses

Data are presented as mean ± standard deviation. Statistical analysis was performed using SPSS 20.0 (IBM, USA). Unpaired two-tailed t-test was used in comparing differences between two groups, and one-way analysis of variance was used in calculating differences among the three groups with the least significant difference test. Statistical significance was set at *P* < 0.05.

## Results

### KD ameliorates HFD-induced adiposity and fatty liver

**Fig. 1:**
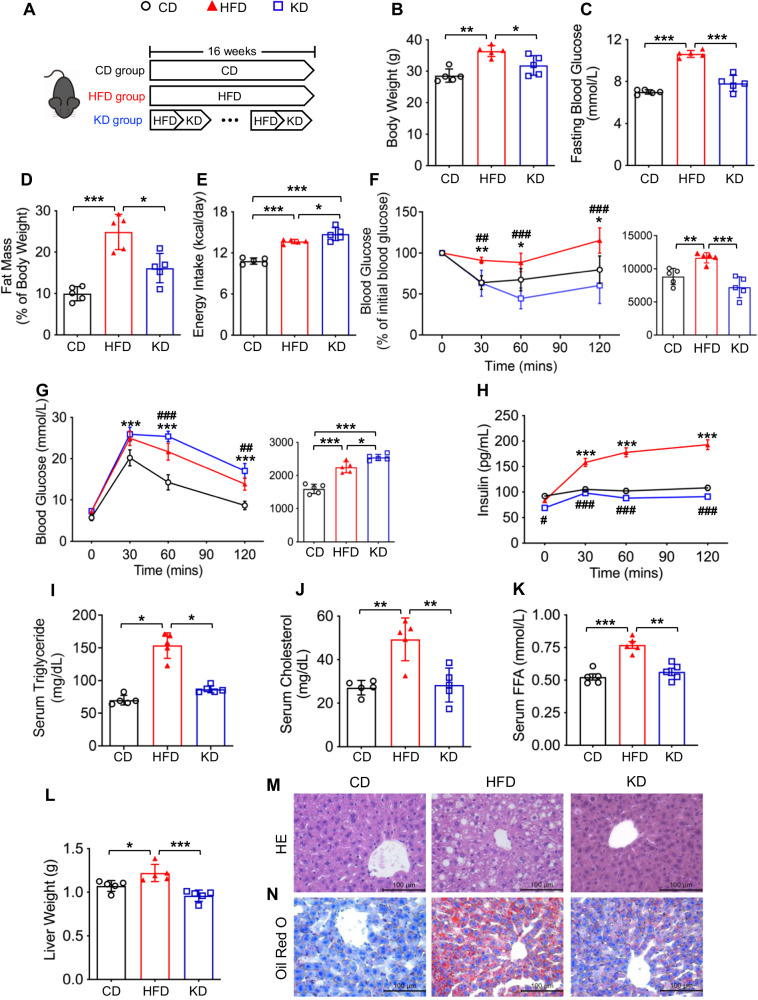
KD ameliorates HFD-induced adiposity and fatty liver. **A** Procedure of the animal experiment. **B** Body weight. **C** Fasting blood glucose. **D** Fat mass-to-body weight ratio. **E** Energy intake. **F** Intraperitoneal insulin tolerance test. **G** Intraperitoneal glucose tolerance test. **H** Intraperitoneal insulin release test. **I** Serum triglyceride level. **J** Serum cholesterol level. **K** Serum free fatty acid level. **L** Liver weight. **M** Hematoxylin and eosin staining of the liver (magnification 400×, scale bar, 100 μm). **N** Oil Red O staining of the liver (magnification 400×, scale bar, 100 μm). CD, chow diet; HFD, high-fat diet; KD, ketogenic diet. n = 5 mice per group. *P < 0.05; **P < 0.01; ***P < 0.001. Line chart: *P < 0.05, **P < 0.01, ***P < 0.001, HFD group *vs* CD group; ^#^*P* < 0.05, ^##^*P* < 0.01, ^###^*P* < 0.001, KD group *vs* HFD group.

To investigate the effect of KD on fatty liver in HFD-induced obese mice, we compared the metabolic characteristics of CD, HFD, and KD groups. After 16 weeks of diet intervention (Fig. 1A), the body weight (31.86 ± 3.12 *vs* 36.44 ± 1.74 g; *P* < 0.05), fasting blood glucose (7.82 ± 0.76 *vs* 10.64 ± 0.33 mmol/L; *P* < 0.001), and fat mass-to-body weight ratio (16.12% ± 3.54% *vs* 24.89% ± 4.26%; *P* < 0.05) of mice in the KD group were significantly lower than those in the HFD group (Fig. 1B–D) despite higher energy intake (14.75 ± 0.94 *vs* 13.66 ± 0.30 kcal/d; *P* < 0.05) (Fig. 1E). Although we did not observe a beneficial effect on glucose tolerance, insulin resistance was remarkably improved, with lower serum insulin levels in the KD group (Fig. 1F–H). Remarkable decrease in circulating lipid profiles, including triglyceride (86.71 ± 5.30 vs 153.63 ± 19.51 mg/dL; *P* < 0.05), total cholesterol (28.36 ± 7.81 *vs* 49.33 ± 9.83 mg/dL; *P* < 0.01), and free fatty acid (0.56 ± 0.06 *vs* 0.77 ± 0.06 mmol/L; *P* < 0.01), were also found in the KD group (Fig. 1I–K). As the liver is one of the major organs contributing to lipid metabolism, we further characterized the liver weight and histological sections of mice fed with different diets. KD reduced the HFD-increased liver weight (0.96 ± 0.06 *vs* 1.22 ± 0.10 g; *P* < 0.001) (Fig. 1L), implying that KD might alleviate HFD-induced hepatic steatosis. The histological results directly showed that KD reduced the HFD-induced lipid accumulation in the liver (Fig. 1M, N). In summary, KD alleviated HFD-induced obesity and partly ameliorated obesity-related metabolic disorders, including hepatic steatosis.

### KD activates hepatic fatty acid oxidation and suppresses lipogenesis

**Fig. 2:**
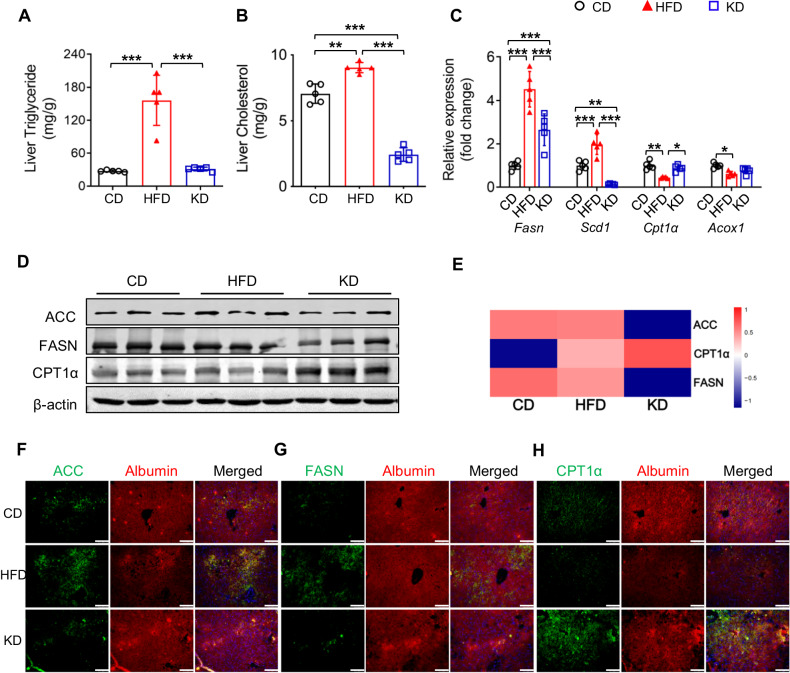
KD activates hepatic fatty acid oxidation and suppresses lipogenesis. **A** Liver triglyceride level. **B** Liver cholesterol level. **C** mRNA expression of genes related to lipid metabolism in the liver. **D** Lipid metabolism related protein levels in the liver. **E** Heatmap of differentially expressed proteins related to lipid metabolism in the liver. Blue, downregulation; red, upregulation. **F**–**H** Immunofluorescence staining of albumin, ACC, FASN, and CPT1α in the liver. Green, ACC **F**, FASN **G**, and CPT1α **H**; red, albumin; blue, DAPI (magnification 200×, scale bar, 100 μm). ACC, acetyl-CoA carboxylase; FASN, fatty acid synthase; CPT1α, carnitine palmitoyltransferase 1α; *Acox1*, acyl-CoA oxidase 1. n = 5 mice per group. *P < 0.05; **P < 0.01; ***P < 0.001.

Next, we measured hepatic lipid levels and lipid metabolism gene expression. Consistently, we found that KD remarkably reduced liver triglyceride (30.99 ± 3.48 vs 156.01 ± 45.47 mg/g; *P* < 0.001) and total cholesterol content (2.43 ± 0.51 *vs* 9.03 ± 0.39 mg/g; *P* < 0.001) compared with HFD (Fig. 2A, B). Using multiple experimental approaches, we demonstrated that the positive lipid metabolic effect of KD was associated with the significant downregulation of lipogenic genes (*Fasn* and *Scd1*) and upregulation of key genes in fatty acid oxidation (*Cpt1α* and *Acox1*) compared with HFD both in transcriptional and translational levels (Fig. 2C–H). Taken together, these results indicated that KD alleviated HFD-induced hepatic steatosis by suppressing lipogenesis and promoting lipid oxidation.

### KD upregulates FGF21 and activates FGF21-KLB signaling in the liver

**Fig. 3:**
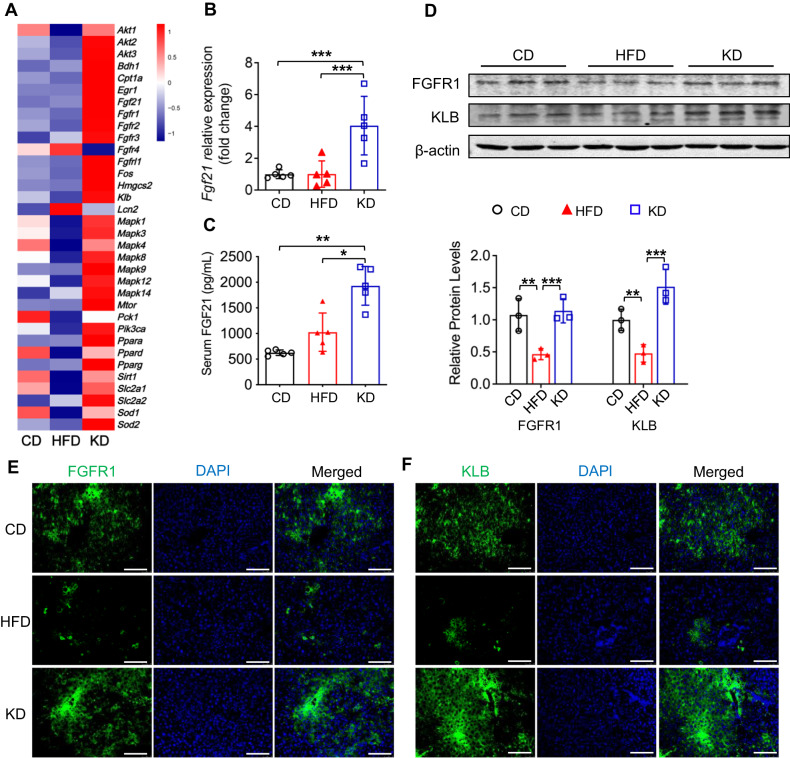
KD upregulates FGF21 and activates FGF21-KLB signaling in the liver. **A** Heatmap showing expression of genes related to FGF21 signaling in the liver. Blue, downregulation; red, upregulation. **B** mRNA expression of *Fgf21* in the liver. **C** FGF21 levels in circulating. **D** FGFR1 and KLB expression in the liver. **E**, **F** Immunofluorescence staining of albumin, FGFR1 and KLB in the liver. Green, FGFR1 **E** and KLB **F**; blue, DAPI (magnification 200×, scale bar, 100 μm). FGF21, fibroblast growth factor 21; FGFR1, fibroblast growth factor receptor 1; KLB, β-klotho. *n* = 5 mice per group. *P < 0.05; **P < 0.01; ***P < 0.001.

To uncover the mechanisms underlying the improvement in hepatic steatosis by a KD, we performed RNA-Seq analysis on mouse livers and found that the mRNA expression profile of *Fgf21* signaling was significantly upregulated, especially *Fgf21*, *Klb*, and *Fgfr1* (Fig. 3A). The downstream genes of *Fgf21* signaling, including *Egr1* and *c-Fos*, showed the same expression trend (Fig. 3A). We further validated the results using qPCR, ELISA, western blotting, and histological analyses of mouse livers. Consistent with the RNA-Seq data, serum FGF21 levels and expression of FGFR1 and KLB were increased in the KD group (Fig. 3B–F). These data suggested a regulatory effect of KD on serum FGF21 levels and FGF21 signaling activation.

### Liver KLB knockdown diminishes the effect of KD on ameliorating metabolic disorders

**Fig. 4:**
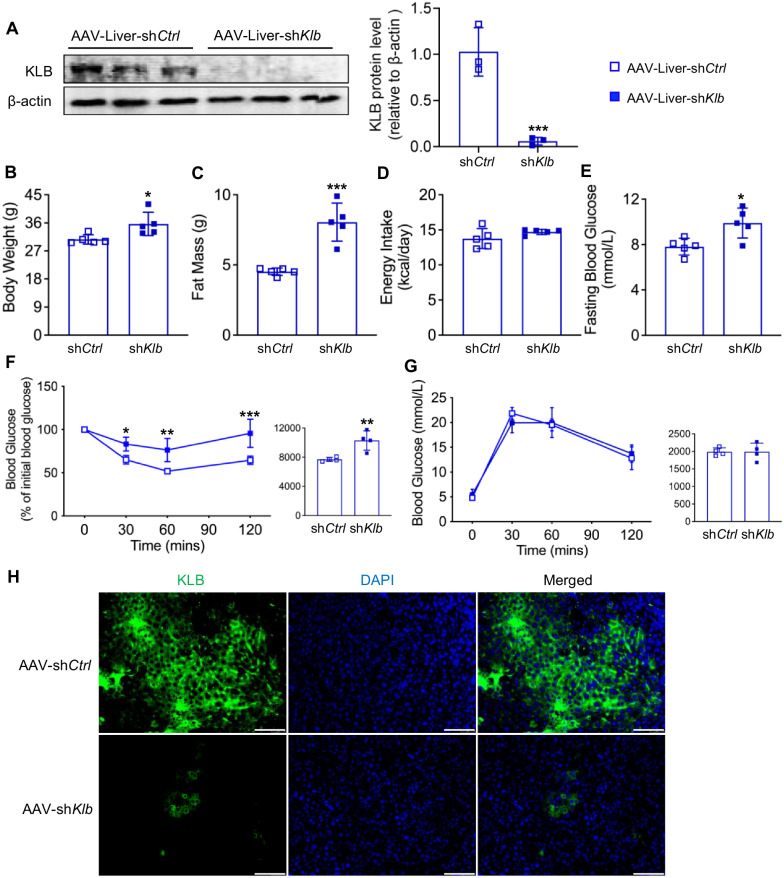
Liver KLB knockdown diminishes the effect of KD on ameliorating metabolic disorders. **A** Liver KLB expression. **B** Body weight. **C** Body fat mass. **D** Energy intake. **E** Fasting blood glucose. **F** Intraperitoneal insulin tolerance test. **G** Intraperitoneal glucose tolerance test. **H** Immunofluorescence staining of albumin and KLB in the liver. Green, KLB; blue, DAPI (magnification 200×, scale bar, 100 μm). KLB, β-klotho. *n* = 4-5 mice per group. *P < 0.05; **P < 0.01; ***P < 0.001.

To further confirm the role of liver FGF21 signaling in the improvement of metabolic disorders induced by a KD, we generated liver KLB knockdown mice using AAV injection. Knockdown efficiency was verified by western blotting and immunofluorescence staining (Fig. 4A, H). Notably, KD-induced improvement of overweight (30.72 ± 1.49 *vs* 35.68 ± 3.74 g; *P* < 0.05), excessive body fat (4.52 ± 0.26 *vs* 8.06 ± 1.36 g; *P* < 0.001), hyperglycemia (7.82 ± 0.73 *vs* 9.90 ± 1.32 mmol/L; *P* < 0.05), and insulin resistance were diminished after KLB knockdown in mouse livers (Fig. 4B, C and E, F). However, liver KLB deficiency had no influence on food intake (13.76 ± 1.42 *vs* 14.72 ± 0.36 kcal/d; *P* > 0.05) and glucose tolerance (Fig. 4D, G). These data revealed the indispensable role of liver KLB in the beneficial hepatic metabolic effects of KD.

### KD-ameliorated hepatic steatosis is dependent on liver FGF21-KLB signaling

**Fig. 5:**
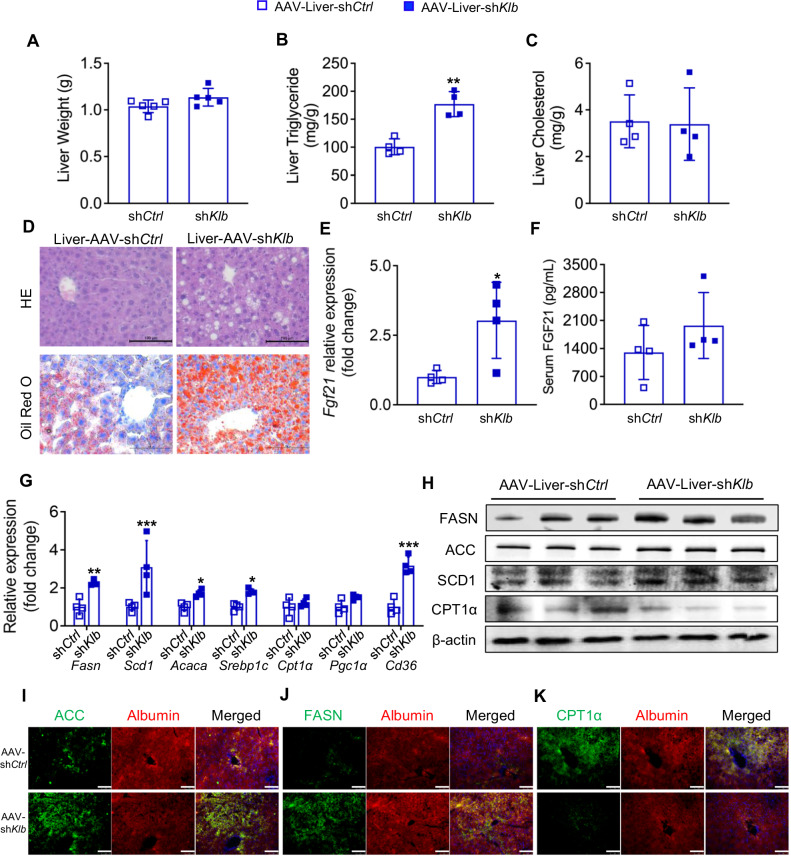
KD-ameliorated hepatic steatosis is dependent on liver FGF21-KLB signaling. **A** Liver weight. **B** Liver triglyceride level. **C** Liver cholesterol level. **D** Hematoxylin and eosin and Oil Red O staining of the liver. **E** Liver *Fgf21* expression. **F** Serum FGF21 level. **G** Expression of genes related to lipid metabolism in the liver. **H** Lipid metabolism related protein levels in the liver. **I**–**K** Immunofluorescence staining of albumin, ACC, FASN, and CPT1α in the liver. Green, ACC **I**, FASN **J**, and CPT1α **K**; red, albumin; blue, DAPI (magnification 200×, scale bar, 100 μm). FGF21, fibroblast growth factor 21; *Fasn*, fatty acid synthase; *Acaca*, acetyl-CoA carboxylase; *Srebp1c*, sterol regulatory element binding protein 1c; *Pgc1a*, peroxisome proliferator-activated receptor gamma coactivator 1-alpha; SCD1, stearyl-coenzyme A desaturase 1. *n* = 4-5 mice per group. *P < 0.05; **P < 0.01; ***P < 0.001.

Although there was no difference in liver weight (1.04 ± 0.07 *vs* 1.14 ± 0.09 g; *P* > 0.05) and total cholesterol content (3.51 ± 1.13 *vs* 3.39 ± 1.56 mg/g; *P* > 0.05) between normal and KLB knockdown mice, liver triglyceride quantification (100.62 ± 14.12 *vs* 177.07 ± 22.41 mg/g; *P* < 0.05) and histological analysis showed that liver KLB knockdown diminished the beneficial effect of KD on alleviating hepatic lipid accumulation (Fig. 5A–D). Although *Fgf21* expression in the liver was upregulated by KLB deficiency, circulating FGF21 levels were unaffected in these mice (Fig. 5E, F). We further examined the transcriptional and translational levels of key hepatic metabolic factors. The results showed that lipogenic genes (*Fasn*, *Scd1*, *Acaca*, and *Srebp1c*) and *Cd36*, which are related to lipid uptake, were significantly upregulated in the livers of KLB knockdown mice without affecting genes related to fatty acid oxidation (Fig. 5G). Similarly, the protein expression of lipogenic proteins (FASN, ACC, and SCD1) was higher, whereas CPT1α was lower in liver KLB knockdown mice (Fig. 5H–K). Thus, liver KLB knockdown impaired the beneficial effect of KD on ameliorating hepatic steatosis, particularly by suppressing lipogenesis, suggesting that FGF21-KLB signaling might be critical for KD-ameliorated hepatic steatosis.

## Discussion

NAFLD is a complex disease for which diet and lifestyle interventions are the main treatment options. Studies have suggested that a KD may ameliorate hepatic steatosis. This study aims to delve deeper into the influence of KD on FGF21 expression and its responsiveness through the FGF21-KLB pathway, underscoring the pivotal role of FGF21-KLB signaling in mediating the KD’s beneficial effects on hepatic steatosis.

Extensive validation in rodent models has established the therapeutic potential of FGF21 for obesity and related metabolic disorders [15, 16, 18–21, 31, 32]. Consistent with prior research, our observations indicated a significant increase in liver FGF21 expression in KD mice [23, 24]. However, it’s noteworthy that heightened endogenous FGF21 levels have been documented in people with obesity, NAFLD, and diabetes [33–35], indicative of a state of FGF21 resistance [36]. This resistance may diminish the sensitivity to endogenous FGF21, potentially limiting the efficacy of low-dose FGF21 treatment [37]. Therefore, enhancing FGF21 sensitivity becomes crucial for achieving its therapeutic effects.

Our investigation revealed that KD elevated hepatic expression levels of KLB and FGFR1 and that KLB plays a critical role in enhancing the binding affinity of FGFR proteins for FGF21. Loss of KLB diminishes these receptors’ responsiveness to FGF21 [26]. Aligned with prior studies [17, 19, 31], we validated that KD upregulates FGF21 and KLB expression, leading to a reduction in hepatic steatosis. Utilizing a liver-specific KLB knockdown mouse model, we further confirmed this association, highlighting the profound impact of KLB deficiency in limiting KD’s improvement of hepatic steatosis, suggesting the indispensability of the liver FGF21-KLB pathway.

In the context of liver metabolism, ACC and FASN proteins play pivotal roles in lipogenesis and are increased during NAFLD progression. Clinical and preclinical studies targeting these proteins have shown significant reductions in hepatic steatosis and NAFLD resolution [38–40]. Our current study demonstrates that KD treatment downregulates both ACC1 and FASN expression at transcript and protein levels while elevating CPT1α expression, which is crucial for β-oxidation in lipid metabolism. However, in the hepatic KLB knockdown mouse model, KD’s effect on the alteration of these genes was nullified. These results suggest that KD suppresses liver lipogenesis, enhances β-oxidation, and ameliorates hepatic steatosis, contingent upon FGF21-KLB signaling. Additionally, we noted that the loss of FGF21-KLB signaling attenuated KD’s effect on decreasing hepatic triglyceride content, but not total cholesterol levels.

Understanding the factors bridging KD and FGF21 remains under exploration. Hepatic FGF21 has been linked as a downstream of peroxisome proliferator-activated receptor alpha (PPARα) [23, 41], activating transcription factor 4 (ATF4) [42] and SIRT1 [43]. KD failed to increase FGF21 expression in PPARα-deficient mice [23], indicating a potential connection between KD and FGF21-KLB signaling. Additionally, the increase in circulating ketones induced by the KD might contribute to its ameliorative effects on metabolic disorders. For example, *Hmgcs2* and *Bdh1*, which are involved in ketogenesis [44], are upregulated by KD, suggesting a novel pathway for KD-ameliorated metabolic disorders. Further research is warranted to explore potential KD-related strategies for the treatment of NAFLD.

However, our study observed limitations of KD. The results of the glucose tolerance test revealed hyperglycemia in the KD group upon glucose stimulation, suggesting potential side effects on β-cell function. Previous research also indicates that KD causes glucose intolerance after 5 weeks and reduces β-cell mass after 22 weeks [45]. Nonetheless, this study contributes to comprehending the mechanisms underlying KD’s therapeutic effects on metabolic disorders, particularly on NAFLD.

By elucidating the crucial involvement of the FGF21-KLB pathway in KD’s beneficial effects on hepatic steatosis, this study sheds light on the intricate interplay among dietary interventions, metabolic signaling pathways, and hepatic lipid metabolism. These findings hold potential for guiding future research and development of targeted therapeutic approaches for managing NAFLD and related conditions.

### Supplementary information

Supplemental Tables

## Data Availability

All data generated or analyzed during this study are included in this published article and its supplementary information files.
