# Supplementary material for: Role of liver FGF21-KLB signaling in ketogenic diet-induced amelioration of hepatic steatosis
Source: Nutr Diabetes. 2024 Apr 12;14:18. doi: 10.1038/s41387-024-00277-3 (PMC11014968; doi:10.1038/s41387-024-00277-3)
Supplement: Supplementary file 1 — Supplemental Tables [file 41387_2024_277_MOESM1_ESM.docx]

**Supplemental Table**

**Table S1. Primers sequences utilized for real-time qPCR.**

| Gene name  (mouse) | Forward sequence (5’-3’) | Reverse sequence (5’-3’) |
| --- | --- | --- |
| *Acaca* | CGAAGGGCTTACATTGCCTA | GGATGTTCCCTCTGTTTGGA |
| *Acox1* | CTGCGGTCCCTTGACCTTTT | TGCATCCATTTCTCCTGCTGA |
| *Cd36* | ATTCCCTTGGCAACCAACCA | CAGCCAGGACTGCACCAATA |
| *Cpt1α* | TCAAGCCAGACGAAGAACATC | TGGTAGGAGAGCAGCACCTT |
| *Fasn* | AAGTTGCCCGAGTCAGAGAA | TGAGGCTGGGTTGATACCTC |
| *Fgf21* | AGCATACCCCATCCCTGACT | AGGAGACTTTCTGGACTGCG |
| *Pgc1α* | TATGGAGTGACATAGAGTGTGCT | CCACTTCAATCCACCCAGAAAG |
| *Scd1* | TGTCTCGGTGTGTGTCGGAGT | TGTACCACTACCTGCCTGCATG |
| *Srebp1c* | CTGGCACTAAGTGCCCTCAAC | GCCACATAGATCTCTGCCAGTGT |
| *β-actin* | TGTGGATCGGTGGCTCCATCCT | AAACGCAGCTCAGTAACAGTCCGC |
